# Structural and Surfacial Modification of Carbon Nanofoam as an Interlayer for Electrochemically Stable Lithium-Sulfur Cells

**DOI:** 10.3390/nano11123342

**Published:** 2021-12-09

**Authors:** Yee-Jun Quay, Sheng-Heng Chung

**Affiliations:** 1 Department of Materials Science and Engineering, National Cheng Kung University, Tainan City 701, Taiwan; yujun990601@gmail.com; 2 Hierarchical Green-Energy Materials Research Center, National Cheng Kung University, Tainan City 701, Taiwan

**Keywords:** lithium-sulfur batteries, high-loading cathode, lean-electrolyte cell, interlayer, electrochemistry

## Abstract

Electrochemical lithium-sulfur batteries engage the attention of researchers due to their high-capacity sulfur cathodes, which meet the increasing energy-density needs of next-generation energy-storage systems. We present here the design, modification, and investigation of a carbon nanofoam as the interlayer in a lithium-sulfur cell to enable its high-loading sulfur cathode to attain high electrochemical utilization, efficiency, and stability. The carbon-nanofoam interlayer features a porous and tortuous carbon network that accelerates the charge transfer while decelerating the polysulfide diffusion. The improved cell demonstrates a high electrochemical utilization of over 80% and an enhanced stability of 200 cycles. With such a high-performance cell configuration, we investigate how the battery chemistry is affected by an additional polysulfide-trapping MoS_2_ layer and an additional electron-transferring graphene layer on the interlayer. Our results confirm that the cell-configuration modification brings major benefits to the development of a high-loading sulfur cathode for excellent electrochemical performances. We further demonstrate a high-loading cathode with the carbon-nanofoam interlayer, which attains a high sulfur loading of 8 mg cm^−2^, an excellent areal capacity of 8.7 mAh cm^−2^, and a superior energy density of 18.7 mWh cm^−2^ at a low electrolyte-to-sulfur ratio of 10 µL mg^−1^.

## 1. Introduction

Commercial lithium-ion batteries use insertion chemical reaction to reversibly release and store lithium ions between the oxide cathode and the graphite anode. The stable crystalline structure of ceramics provides high cycle stability and underpins the successful development of the prosperous lithium-ion battery market [1,2]. In mature lithium-ion technology, the charge-storage capacities of lithium-ion battery cathodes have almost reached their theoretical values. Moreover, it has been proven that the crystalline structures of the oxide cathodes limit the full insertion/extraction of lithium ions. Thus, a bottleneck constrains the increase of battery capacity [2,3,4]. To meet the demands of the continuously growing global energy-storage market, the development of next-generation rechargeable batteries requires novel cathode materials featuring a higher charge-storage capacity and long-term electrochemical stability [4,5,6]. Among the newly developed battery chemistries, lithium–sulfur batteries use sulfur as the cathode material to generate a high theoretical charge-storage capacity of 1672 mAh g^−1^ from a conversion battery chemical reaction, which enables the device to deliver a high energy density of 2600 Wh kg^−1^ and attain high electrochemical efficiency. Moreover, the inexpensive sulfur is nontoxic and naturally abundant. Therefore, great attention is given to lithium–sulfur cells because of their electrochemical and material properties [7,8,9,10,11].

However, novel technologies often bring new challenges. The fast progress in lithium-sulfur batteries has encountered intrinsic material challenges resulting from the poor charge-transfer nature of solid-state sulfur and sulfides as well as the formation and diffusion of liquid-state polysulfides [8,9,10,11,12]. The progress in lithium-sulfur batteries has also been hindered by extrinsic electrochemical limitations encountered in building high-energy-density cathodes with enough active material in a lean-electrolyte cell [13,14,15,16]. In lithium-sulfur battery chemistry, the active material forms solid-state materials, such as sulfur and lithium sulfides at the full charge and discharge stages, respectively. Both solid-state active materials are good insulators, which limits the electrochemical utilization and reversibility of the active material during redox reactions [10,12]. The solid-state active materials convert to liquid-state lithium polysulfides at the intermediate stages of discharging and charging the cell. Lithium polysulfides are soluble in an ether-based liquid electrolyte. The dissolved polysulfides readily diffuse out from the sulfur cathode region and move uncontrollably across the whole cell. The irreversible polysulfide diffusion leads to the rapid loss of the active material and the corresponding degradation of the electrodes and the electrolyte, which results in poor cyclability and unstable discharge/charge efficiency [12,13]. As a result of the high resistance brought about by the active material in the solid state and the irreversible diffusion problem of the active material in the liquid state, the lithium-sulfur literature usually reports the performance of devices utilizing a sulfur loading less than 1–2 mg cm^−2^ and a sulfur content lower than 60 wt% in a cell with a high amount of liquid electrolyte (i.e., an electrolyte-to-sulfur ratio of over 20 µL mg^−1^) [4,13,14,15,16]. Unfortunately, such a cell composition hinders the development and investigation of high-energy-density lithium-sulfur cells, which require high-loading sulfur cathodes to achieve high electrochemical utilization of sulfur in a lean-electrolyte condition [16,17,18,19].

In this study, we design, investigate, and modify the battery configuration by considering the conversion electrochemical reaction of the lithium–sulfur battery cathode. We adopt a conductive porous carbon substrate as the carbon-nanofoam interlayer between the sulfur cathode and the separator [4,19,20,21,22]. The interlayer utilizes its conductive carbon network to improve the cathode conductivity, which results in an improved electrochemical utilization of sulfur [12,20,21,22]. During cycling, the interlayer offers a tortuous and porous network for reducing the migration of liquid-state polysulfides and subsequently trapping them. The trapped polysulfides are stabilized within the carbon interlayer and the cathode region so that the active material retains a high electrochemical activity. This results in a high electrochemical reversibility and cycle stability [20,21,22]. Thus, our findings demonstrate that the carbon-nanofoam interlayer allows high-loading sulfur cathodes to attain sulfur loadings and content of 4–8 mg cm^−2^ and 70 wt%, respectively, and to exhibit high discharge capacities of 1087–1131 mAh g^−1^ at the C/20 and C/10 rates for a long cycle life of 200 cycles at a low electrolyte-to-sulfur ratio of 10 µL mg^−1^. The resulting high-loading sulfur cathodes achieve high electrochemical utilization and, therefore, exhibit a high areal capacity and energy density of 8.7 mAh cm^−2^ and 18.7 mWh cm^−2^, respectively. We further study the material modification of the carbon interlayer with surface coatings by adopting a polysulfide-trapping MoS_2_ layer [23,24,25] and an electron-transferring graphene layer [26,27,28]. The additional layers aid the exploration of the importance of the battery-configuration design and the material properties toward the electrochemical characteristics of the high-loading sulfur cathode.

## 2. Materials and Methods

### 2.1. Materials and Chemical Characterization

Microstructure inspection and elemental mapping analyses were performed with an SU-8000 field-emission scanning electron microscope (Hitachi, Tokyo, Japan) and the equipped XFlash 5010 energy-dispersive X-ray spectrometer (Bruker, Billerica, MA, USA), respectively. Physical adsorption/desorption analysis was carried out with an Autosorb iQ MP/MP gas sorption analyzer (Anton Paar, Austria) at 77 K at P/P_0_ = 10^−5^ to 10^0^. Specific surface area and porosity analyses (e.g., the total pore volume and average pore size) were performed based on the Brunauer-Emmett-Teller theory and T-plot analysis. The pore size distribution was analyzed according to Barrett-Joyner-Halenda theory, density functional theory, and Horvath-Kawazoe theory.

Surface characterization and bonding energy analysis were conducted with a PHI 5000 VersaProbe III X-ray photoelectron spectrometer (Ulvac-Phi, Chanhassen, MN, USA). Chemical functional groups were detected using infrared spectroscopy performed on a Nicolet iS50 Fourier-transform infrared spectrometer (Thermo Fisher, Waltham, MA, USA). Carbon information was characterized by Raman spectroscopy conducted on a Labram HR Micro-Raman & Micro-PL spectrometer (Jobin Yvon, Paris, France) using a 532 nm laser excitation.

### 2.2. Electrochemical and Cell Performance Characterization

Electrochemical and lithium-sulfur battery performance analyses were conducted using a BCS-800 series battery-test instrument (Biologic Science Instruments, France). The lithium-sulfur cells with various configurations (i.e., with the carbon-nanofoam interlayer, the MoS_2_-coated carbon-nanofoam interlayer, the graphene-coated carbon-nanofoam interlayer, and the reference cathode) were analyzed with a voltage window of 1.8–2.8 V at rates of C/20 and C/10 to investigate the discharge/charge voltage profiles, electrochemical polarization, long-term cyclability, and charge-storage capacity. CV analysis was performed at 0.01 mV s^−1^ for five scans.

The carbon nanofoam was a commercial carbon-paper substrate (High Tech Material Solutions, Auburn, WA, USA). The MoS_2_-coated carbon nanofoam and the graphene-coated carbon nanofoam were modified by chemical vapor deposition to generate a thin nanolayer of MoS_2_ and graphene on the carbon nanofoam (carried out at the Core Facility of National Cheng Kung University). A MoO_3_ precursor film with a thickness of 10 nm was grown on the carbon nanofoam at 300 °C and 2 × 10^−5^ torr and underwent sulfurization by H_2_S at 700 °C for 60 min, generating the MoS_2_-coated carbon nanofoam. A gas mixture of Ar (1500 sccm), H_2_ (200 sccm), and diluted CH_4_ (5 sccm) was applied to the carbon nanofoam at 900 °C for 150 min to form the graphene-coated carbon nanofoam.

The lithium-sulfur cells were prepared with a high-loading sulfur cathode (12 mm in diameter), the carbon-nanofoam interlayer (12 mm in diameter), a polypropylene membrane (Celgard, 19 mm in diameter), and a metallic lithium counter electrode (Sigma-Aldrich, 14 mm in diameter). Specifically, the sulfur cathode was prepared by mixing 70 wt% sulfur, 15 wt% Super P carbon, and 15 wt% polyvinylidene fluoride binder in N-methyl-2-pyrolidone. 

The well-mixed paste was then tape-casted on an aluminum-foil current collector and dried in a convection oven at 50 °C for 24 h. The electrolyte contained 1.85 M lithium bis(trifluoromethanesulfonyl)imide (Acros Organics) and 0.1 M lithium nitrate (Acros Organics) dissolved in 1,3-dioxolane/1,2-dimethoxyethane solvent. The assembled cells held high sulfur loadings of 4 and 8 mg cm^−2^ at a low electrolyte-to-sulfur ratio of 10 µL mg^−1^.

## 3. Results and Discussion

### 3.1. Material Characterization of the Carbon Nanofoams

**Figure 1:**
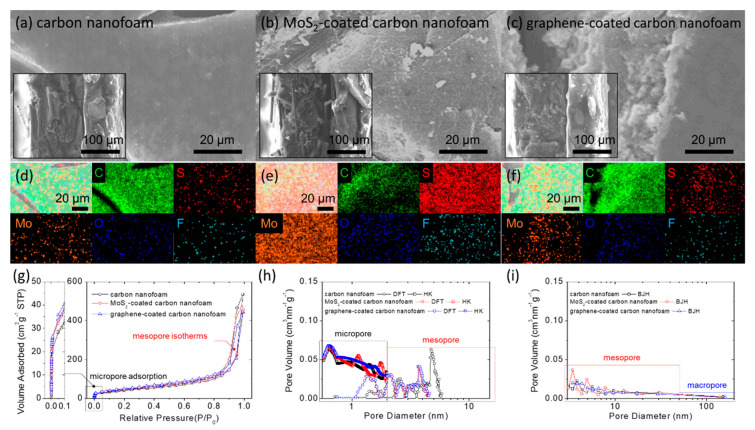
Material characterization: microstructural inspection of the (**a**) carbon nanofoam, (**b**) MoS_2_-coated carbon nanofoam, and (**c**) graphene-coated carbon nanofoam by field-emission scanning electron microscopy (insets are the cross-sectional inspection); elemental mapping results of the (**d**) carbon nanofoam, (**e**) MoS_2_-coated carbon nanofoam, and (**f**) graphene-coated carbon nanofoam by energy-dispersive X-ray spectroscopy; porosity analysis with (**g**) nitrogen adsorption/desorption isotherms, (**h**) pore size distribution calculated with density functional theory (DFT) and Horvath–Kawazoe (HK) theory, and (**i**) Barrett-Joyner-Halenda (BJH) pore size distribution.

Figure 1a–c shows the scanning electron microscopy microstructural analysis of the carbon nanofoam and its two surfacial modification derivatives with the MoS_2_ coating and the graphene coating processed by chemical vapor deposition. Surface microstructural inspection reveals that the carbon-nanofoam substrate has a continuous carbon nanofiber as the conductive skeleton, on which nanoporous carbon substrates are tightly attached (Figure 1a). 

With modification by MoS_2_ and graphene coating layers, the surface morphologies of the MoS_2_-coated carbon nanofoam and the graphene-coated carbon nanofoam display obvious surface coverage (Figure 1b,c). The chemical composition is analyzed by elemental mapping through energy-dispersive X-ray spectroscopy, the results of which (Figure 1d–f and Table 1) confirm the obvious elemental carbon signals (marked as green) in the carbon nanofoam and the graphene-coated carbon nanofoam. Distinct elemental molybdenum and sulfur signals (marked as orange and red, respectively) brought about by the MoS_2_ coating can be detected from the MoS_2_-coated carbon nanofoam.

Figure 1g–i summarizes the surface and porosity analysis of the carbon-nanofoam samples. The three carbon nanofoams depict similar mixed IUPAC types I and IV isotherms, showing micropore adsorption at the low relative pressure region (left plot in Figure 1g) and a mesopore hysteresis loop at the high relative pressure region (right plot in Figure 1g) [29]. 

The pore-size distribution analysis indicates the appearance of both micropores and small-sized mesopores with diameters of less than 5 nm (Figure 1h,i). The specific surface area and total pore volume of the carbon nanofoam, the MoS_2_-coated carbon nanofoam, and the graphene-coated carbon nanofoam are 146, 165, and 171 m^2^ g^−1^ and 0.86, 0.68, and 0.64 cm^3^ g^−1^, respectively. After the surface modification, the increase in surface area and the decrease in total pore volume suggest that the deposited coating layers generate slit nanopores and cover the surface of the carbon nanofoam. 

The average pore size decreases from 3.83 nm for the carbon nanofoam to 3.41 and 3.42 nm for the MoS_2_-coated carbon nanofoam and the graphene-coated carbon nanofoam, respectively, and thus agrees with the surface-area and pore-volume trends. As a result, the microstructural analysis affirms the functional decoration on the surface of the carbon nanofoam with either MoS_2_ or graphene layers. However, the matrix substrates of the modified carbon nanofoams are controlled with similar physical material characteristics.

### 3.2. Chemical Analysis of Carbon Nanofoam

**Figure 2:**
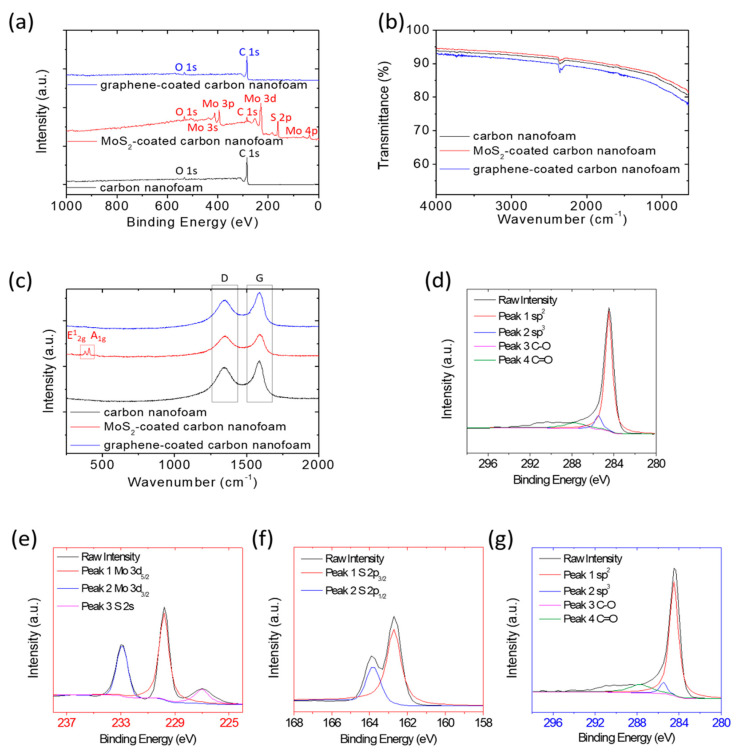
Chemical analysis: (**a**) X-ray photoelectron spectra, (**b**) Fourier-transform infrared spectra, and (**c**) Raman spectra; X-ray photoelectron characteristic-peak analysis of (**d**) carbon nanofoam, (**e**,**f**) MoS_2_-coated carbon nanofoam, and (**g**) graphene-coated carbon nanofoam.

Figure 2 summarizes the X-ray photoelectron spectroscopy (Figure 2a), Fourier-transform infrared spectroscopy (Figure 2b), and Raman spectroscopy (Figure 2c) results for the chemical analysis of the carbon nanofoams with and without surfacial modifications. The X-ray photoelectron survey spectra confirm the sharp difference between, on one hand, the carbon nanofoam and the graphene-coated carbon nanofoam, both of which feature a strong carbon peak, and on the other hand, the MoS_2_-coated carbon nanofoam, which is characterized by molybdenum and sulfur peaks (Figure 2a). As a reference, the oxygen signal at 536 eV brought about by the O 1s peak is likely to result from the atmosphere.

Subsequently, we analyze the X-ray photoelectron elemental characteristic peaks of the samples. The carbon nanofoam and the graphene-coated carbon nanofoam both show C 1s characteristic peaks (Figure 2d,g), including sp^2^ hybridization from the C=C bond (284.5 eV), sp^3^ hybridization from the carbon clusters’ C-C bonds (285.5 eV), C-O bonds (286.7 eV), and C=O bonds (287.7 eV) [26,27,28,30,31]. In contrast, the MoS_2_-coated carbon nanofoam indicates Mo 3d and S 2s characteristic peaks, featuring Mo 3d_3/2_ (232.9 eV) and Mo 3d_5/2_ (229.2 eV), along with S 2s (226.1 eV) arising from S^2−^. 

The S 2p spin orbits are separated into S 2p_3/2_ (162.7 eV) and S 2p_1/2_ (163.8 eV), also arising from S^2−^, based on the configuration of sulfur in MoS_2_ (Figure 2e,f) [23,24,25]. The chemical analysis affirms the carbon nanofoam as a pure carbon matrix. Furthermore, the successful modification of the carbon nanofoam with the graphene coating to form the graphene-coated carbon nanofoam and with the MoS_2_ coating to generate the MoS_2_-coated carbon nanofoam is also affirmed.

Figure 2b,c displays the results of surface chemical analysis using, respectively, Fourier-transform infrared spectroscopy and Raman spectroscopy. The infrared spectra indicate that the carbon nanofoams show no obvious differences in the surface functional groups before and after functional coating with MoS_2_ and graphene. This conforms with the lack of any other changes in the chemical properties or the formation of any impurities during the fabrication processes (Figure 2b). 

In consideration of the almost unchanged surface chemical composition, the Raman spectrum is measured to analyze the carbon nanofoams by characterizing their degree of graphitization. All carbon-nanofoam samples show the disordered carbon sp^3^ band due to the disordered asymmetric vibration at 1350 cm^−1^ and the graphitic carbon sp^2^ band due to the in-plane stretch at 1580 cm^−1^ (Figure 2c). The MoS_2_-coated carbon nanofoam displays two additional characteristic E^1^_2g_ and A_1g_ peaks at 381 and 406 cm^−1^, which are associated with the in-plane and out-of-plane lattice vibrations of MoS_2_, respectively [23,24,25]. 

The intensity ratios between the disordered carbon band and the graphitic carbon band are 0.84, 0.94, and 0.78 for the carbon nanofoam, the MoS_2_-coated carbon nanofoam, and the graphene-coated carbon nanofoam, respectively. The carbon nanofoam and the graphene-coated carbon nanofoam display a strong graphitic carbon sp^2^ band (i.e., a relatively low ratio), which might result from the highly graphitized carbon backbone and conductive graphene coating [12,32,33]. 

The chemical analysis shows that the carbon nanofoams decorated with MoS_2_ and graphene only contain surface functional coatings, with no additional changes in the carbon matrix. These two modification coating layers allow the materials to serve as excellent platforms to explore the effect of the cathode configuration and material modification on the electrochemical performance of lithium-sulfur battery cathodes.

### 3.3. Electrochemical Analysis of Carbon Nanofoam

**Figure 3:**
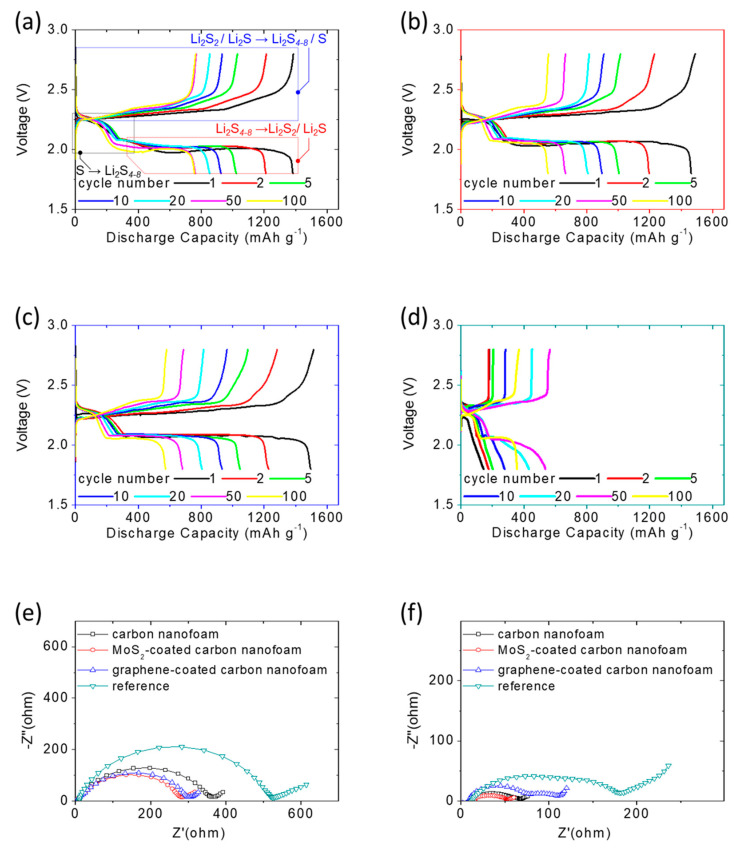
Electrochemical analysis: discharge/charge voltage profiles of the sulfur cathodes at the C/20 rate with (**a**) the carbon-nanofoam interlayer, (**b**) the MoS_2_-coated carbon-nanofoam interlayer, (**c**) the graphene-coated carbon-nanofoam interlayer, and (**d**) the reference cathode; and impedance analysis of the sulfur cathodes with carbon-nanofoam interlayers (**e**) before and (**f**) after 100 cycles.

Figure 3a–d shows the discharge/charge voltage profiles of the lithium-sulfur cells at the C/20 rate with different carbon-nanofoam interlayers, i.e., the carbon-nanofoam cathode (marked as the black box), the MoS_2_-coated carbon-nanofoam cathode (marked as the red box), and the graphene-coated carbon-nanofoam (marked as the blue box). The functional coating layers are configured to face the sulfur cathode to investigate their functional performances. A cell with the conventional lithium-sulfur cell configuration was prepared as the reference cathode, shown by the green box. The experimental cells and reference cell all hold a fixed high sulfur loading and content of 4 mg cm^−2^ and 70 wt%, respectively [4,13,14,15].

In Figure 3a–d, the as-prepared cells are initially discharged to 1.8 V and subsequently charged to 2.8 V as the cut-off discharge and charge voltage values, respectively, at the C/20 rate. The discharge/charge voltage profiles depict two distinguishable discharge plateaus. The upper discharge plateau starting at 2.3 V represents the reduction conversion of solid-state elemental sulfur to liquid-state long-chain lithium polysulfides featuring the chemical formula Li_2_S_x_ with x = 4–8. The formation of lithium polysulfides at this stage generates dissolved polysulfides, which have a high reactivity and function as the catholyte, in the liquid electrolyte. 

This contrasts with a conventional sulfur cathode, in which it is common to observe the rapid diffusion and uncontrollable migration of the dissolved polysulfides out of the cathode, which causes the loss of active material, degradation of the cathode, and irreversible capacity loss [4,12,13]. The lower discharge plateau starting at 2.1 V is associated with the subsequent conversion from the liquid-state lithium polysulfides to solid-state lithium sulfides as a mixture of Li_2_S_2_/Li_2_S. 

The liquid-to-solid conversion and material reduction reaction, along with the redeposition of the diffusing polysulfides on the surface of electrodes as the inactive zone with high resistance, are sluggish processes [10,13]. In our work, all of the high-loading sulfur cathodes with different types of carbon-nanofoam interlayers show approximately unchanged upper discharge plateaus and overlapping lower discharge plateaus during 100 continuous cycles, demonstrating the superior polysulfide retention and outstanding reaction capability, respectively (Figure 3a–c). 

In contrast, the reference sulfur cathode with a conventional cathode configuration suffers from the inefficient and unstable electrochemical reaction of lithium-sulfur batteries, showing a low peak discharge capacity of 559 mAh g^−1^ and requiring an activation process of 20 cycles before reaching the maximum electrochemical utilization of the high amount of insulating sulfur (Figure 3d). During the charge state, the two overlapping charge plateaus at 2.2–2.3 V correspond to two reversible oxidation conversions: (1) from solid-state lithium sulfides to lithium polysulfides (Li_2_S_4–8_) and (2) from Li_2_S_4–8_ to a Li_2_S_8_/sulfur mixture [12,30,31].

The analysis of the discharge/charge voltage profiles demonstrates that the adoption of the carbon-nanofoam interlayer endows the high-loading sulfur cathode with (1) strong polysulfide retention because the tortuous and porous network of the carbon nanofoam inhibits the rapid leakage of liquid polysulfides out of the cathode, and (2) high reaction kinetics because the conductive network of the carbon-nanofoam interlayer enables rapid electron transfer in the cathode, as shown in the electrochemical impedance data. 

The impedance data demonstrated the significant decrease of the cathode resistance of the cell with the carbon-nanofoam interlayer (Figure 3e), which also maintained a low cell impedance after cycling (Figure 3f). Moreover, the polysulfides trapped in the cathode region of the cells function as a catholyte because of the strong reactivity of dissolved polysulfides. They further improve the electrochemical utilization of the high-loading sulfur cathode, resulting in enhanced high charge-storage capacity values of 1381–1484 mAh g^−1^ [4,12,22]. 

Subsequently, we investigate the voltage values of the long, flat lower discharge plateau and the charge plateau to characterize the electrochemical polarization of the cells. Figure 3a–d displays the relatively weak polarization of the cells with carbon-nanofoam interlayers. This demonstrates that the adoption of the carbon-nanofoam interlayer mitigates the increasing large-voltage hysteresis originally brought about by the continuous diffusion of the liquid-state polysulfides and the repeated deposition of insulating solid-state active materials on the cathode.

**Figure 4:**
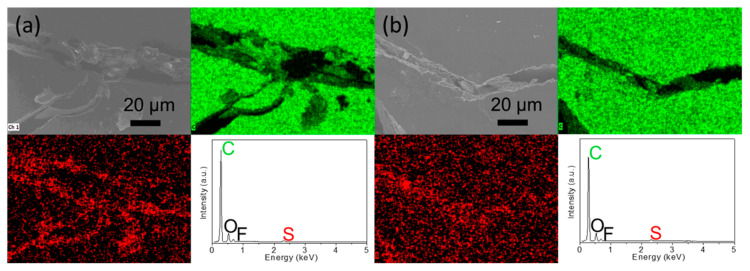
Electrochemical analysis: microstructural inspection and elemental mapping results of the inserted carbon paper for the polysulfide diffusion analysis: (**a**) the side facing cathode and (**b**) the side facing anode.

We further design an analytical cell with an additional carbon paper to examine the polysulfide-trapping capability of the modified cell with the carbon-nanofoam interlayer. Specifically, the designed cell has a sulfur cathode, a carbon-nanofoam interlayer, a polypropylene membrane, additional carbon paper, another polypropylene membrane, and a metallic lithium counter electrode. Figure 4a,b displays the microstructural and elemental analysis of the cycled additional carbon paper facing the cathode side and the anode side, respectively. 

Our analytical results confirm almost no sulfur-related morphology and elemental signals that can be detected, demonstrating the excellent polysulfide retention and related high chemical stability. This provides solid evidence for the advantages of using the carbon-nanofoam interlayer to improve the active-material utilization and electrochemical stability [20,21,22]. Furthermore, it is found that the carbon-nanofoam interlayers modified with the MoS_2_ and graphene surface coatings display similar discharge and charge performances to the unmodified coatings, implying similar improvements in the battery electrochemistry (Figure 3b,c).

**Figure 5:**
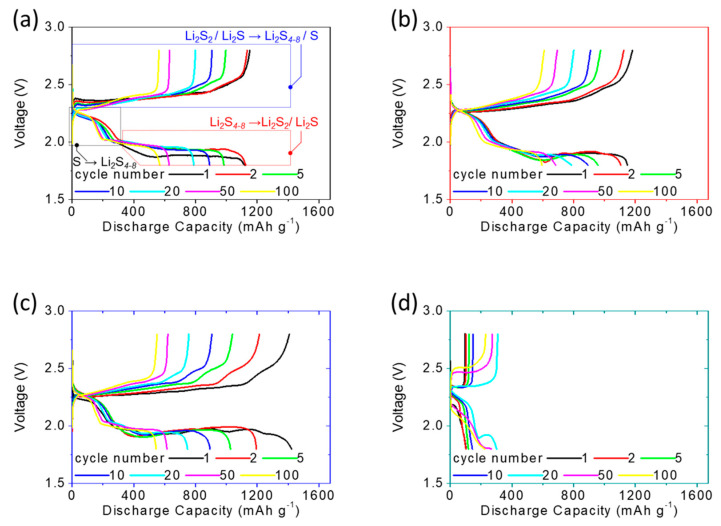
Electrochemical analysis: discharge/charge voltage profiles of the sulfur cathodes at the C/10 rate with (**a**) the carbon-nanofoam interlayer, (**b**) the MoS_2_-coated carbon-nanofoam interlayer, (**c**) the graphene-coated carbon-nanofoam interlayer, and (**d**) the reference cathode.

To support the above-mentioned performance comparison, Figure 5 summarizes the discharge/charge voltage profiles of the cells at the C/10 rate. At the cycling rate of C/10, the cells with various carbon-nanofoam interlayers display similar high electrochemical performances. The overlapping discharge and charge curves indicate the superior cycle stability, while the similar discharge and charge capacity values represent the high electrochemical reversibility of the sulfur cathode, which is contributed to by these carbon-nanofoam interlayers [4,10,12]. 

However, the voltage hysteresis caused by the high current density increases when the carbon-nanofoam interlayers are decorated with functional coatings. This might be associated with the covering of the carbon nanofoam by the functional coatings as revealed in the physicochemical analysis. Although the coating layers contribute to a slight increase in the surface area and additional electrochemical functions, they block access to the nanopores, in which polysulfides would be trapped and stabilized to serve as the electrochemical catholyte. 

Thus, the MoS_2_-coated carbon-nanofoam and graphene-coated carbon-nanofoam interlayers provide limited improvements, which suggests that the modification of the cell configuration with the carbon-nanofoam interlayer affords a major improvement in conversion lithium–sulfur battery cathodes. In sharp contrast with the modified cathode, the reference cathode cannot normally cycle with such a high amount of sulfur. At a high current density, the insulating sulfur and the serious polysulfide diffusion in the high-loading sulfur cathode pose challenges in developing high-energy-density lithium–sulfur cells with the conventional cathode configuration—namely, the high electrochemical polarization, the inefficient electrochemical utilization, and the poor reaction stability [13,14,15,16,32,33,34].

**Figure 6:**
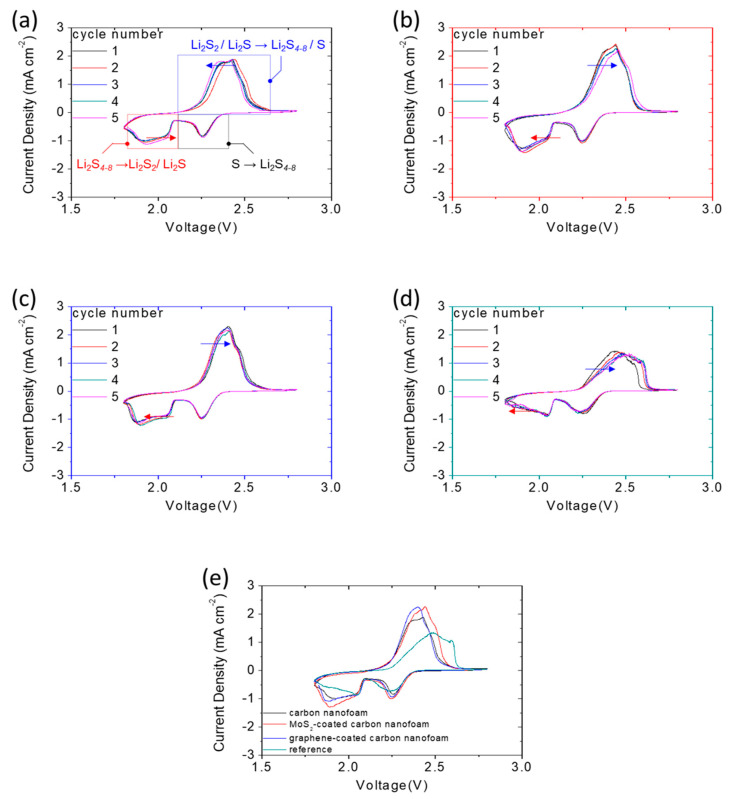
Electrochemical analysis: cyclic voltammetry (CV) analysis of the sulfur cathodes with (**a**) the carbon-nanofoam interlayer, (**b**) the MoS_2_-coated carbon-nanofoam interlayer, (**c**) the graphene-coated carbon-nanofoam interlayer, (**d**) the reference cathode, and (**e**) various carbon-nanofoam interlayers.

Additional electrochemical evidence is shown in Figure 6, which summarizes the cyclic voltammetry (CV) curves of the high-loading sulfur cathodes with and without the carbon-nanofoam interlayers at a 0.01 mV s^−1^ scanning rate. The CV curves of the cells with the carbon-nanofoam interlayers agree with the discharge and charge voltage profiles, depicting the stable and reversible redox reactions (Figure 6a–c). The IR drop shown in the cell might result from the use of the high amount of sulfur and the reducing amount of electrolyte, while causing limited impacts on the redox scans. 

Among these optimal cathode configurations, the cathode with the carbon nanofoam displays the highest electrochemical reversibility, featuring the shift of the reduction peaks toward high voltages (marked by a red arrow), while the oxidation peaks shift toward low voltages (marked by a blue arrow) [7,12,13]. In stark contrast, the reference sulfur cathode shows a decrease in the electrochemical reversibility and an increase in the polarization. Moreover, it suffers a low electrochemical utilization (Figure 6d,e).

### 3.4. Lithium-Sulfur Cell Performance of Carbon Nanofoam

**Figure 7:**
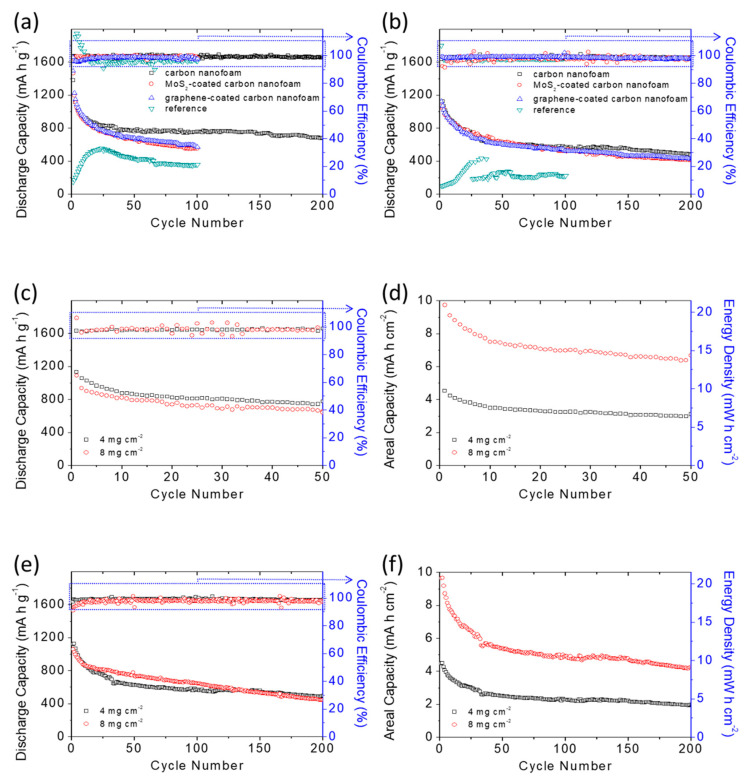
Battery performance analysis: cyclability of the sulfur cathodes with various carbon-nanofoam interlayers at (**a**) the C/20 rate and (**b**) the C/10 rate; battery performance and areal capacity and energy density analysis of the high-loading sulfur cathodes with the carbon-nanofoam interlayer at (**c**,**d**) the C/20 rate and (**e**,**f**) the C/10 rate.

Figure 7a,b shows the cycling performance of the high-loading sulfur cathodes with and without the carbon-nanofoam interlayers at the C/20 and C/10 rates for 200 cycles. In Figure 7a, the cells with the carbon-nanofoam interlayer, the MoS_2_-coated carbon-nanofoam interlayer, the graphene-coated carbon-nanofoam interlayer, and the reference cathode show peak discharge capacity values of 1381, 1462, 1484, and 558 mAh g^−1^, respectively, and reversible discharge capacity values of 757, 552, 572, and 357 mAh g^−1^, respectively, after 100 cycles at the C/20 rate. 

The cycling performances demonstrate that the modified cathode configuration with the carbon-nanofoam interlayer results in a significant improvement in the electrochemical utilization from 33% to 82–89%. Moreover, the carbon-nanofoam interlayers, which accelerate the charge transfer, solve the problem of inefficient activation of the high-loading sulfur reference cathode. Among these interlayers, the cell with the carbon-nanofoam interlayer retains the highest reversible discharge capacity and the best cycle stability after the long period of cycling with an extended cycle life toward 200 cycles.

When the cycling rate increases to C/10, Figure 7b shows that the cells with the carbon-nanofoam interlayer, the MoS_2_-coated carbon-nanofoam interlayer, and the graphene-coated carbon-nanofoam interlayer all display high peak and reversible (given in parentheses) discharge capacities after 200 cycles of 1125 (500), 1109 (413), and 1115 (428) mAh g^−1^, respectively. However, the reference cathode shows low peak and reversible capacities of 432 and 222 mAh g^−1^, respectively, after 100 cycles. The cells with the carbon-nanofoam interlayers again demonstrate the highest electrochemical utilization and stability at this high cycling rate. 

High-rate testing also confirms the significant improvement in the cell performance with the use of the carbon-nanofoam interlayer. The carbon-nanofoam interlayer again outperforms the modified samples. In contrast, the reference cathode encounters low utilization of 25% and basically fails after 40 cycles. The performance metrics on each type of interlayer, and the references are summarized in Table 2 as a reference. In addition to the cell capacities, the Coulombic efficiency agrees with the capacity performance and shows enhancement when the carbon-nanofoam interlayer is applied in the cell. 

The cell without the carbon-nanofoam interlayer suffers an unstable Coulombic efficiency during the initial activation cycling and faces a relative low efficiency in the subsequent cycles. In contrast, the carbon-nanofoam interlayers function as the additional upper current collector in the cell, which offers fast electron transfer and lithium-ion diffusion. Thus, the modified cells have high and stable Coulombic efficiency in the cycling-performance analysis.

On the basis of the electrochemical characteristics analyzed above, we optimize the cell with the carbon-nanofoam interlayer and attain an increased (doubled) sulfur loading of 8 mg cm^−2^ and a decreased electrolyte-to-sulfur ratio of 10 µL mg^−1^ to evaluate the feasibility of our cell design. Figure 7c shows that the high-loading sulfur cathode with the carbon-nanofoam interlayer attains a high discharge capacity of 1087 mAh g^−1^, approaches 65% utilization, and features a high electrochemical reversibility at the C/20 rate in the lean-electrolyte lithium-sulfur cell. 

The development of a high-loading sulfur cathode with a high electrochemical utilization is the key for the lithium–sulfur battery to attain a high energy density. Accordingly, we analyze the specific areal capacity and energy density of the cell, as shown in Figure 7d. The cells featuring the high-loading sulfur cathode and lean-electrolyte conditions attain high specific areal capacity and energy density of 8.7 mAh cm^−2^ and 18.7 mWh cm^−2^, respectively. At the C/10 rate, the same high-loading sulfur cathode realizes a long cycle life of 200 cycles and a high discharge capacity of 1057 mAh g^−1^, which corresponds to high areal capacity and energy density of 8.4 mAh cm^−2^ and 18.2 mWh cm^−2^, respectively (Figure 7e,f). 

Therefore, the application of the carbon-nanofoam interlayers results in outstanding performance improvements and outperforms the values needed for powering electric vehicles (i.e., 2–4 mAh cm^−2^) and for serving as an alternative to the conventional lithium-ion battery cathode (i.e., 11 mWh cm^−2^) [4,9,10,11,12]. Moreover, a comparison with other state-of-the-art works in high sulfur loading systems suggests the carbon-nanofoam interlayer that could attain a good balance in the battery performances (e.g., high amount of sulfur and low amount of electrolyte) and electrochemical characteristics (e.g., discharge capacity, areal capacity, and cycle life, etc.). 

Thus, the comparison demonstrates the cell configuration of adopting the carbon-nanofoam interlayer and the related progresses in references as possible cell development in future lithium-sulfur research (Table 3) [35,36,37,38,39,40,41,42,43,44,45].

## 4. Conclusions

In summary, we designed, modified, and investigated the effect of applying a carbon-nanofoam interlayer in lean-electrolyte lithium–sulfur cells with high-loading sulfur cathodes. The carbon-nanofoam interlayer inserted between the sulfur cathode and the separator possessed a tortuous and porous carbon network for decreasing the migration of liquid polysulfides and increasing the transfer of electrons and lithium ions in the high-loading sulfur cathodes. 

As a result, lithium-sulfur cells with a carbon-nanofoam interlayer enabled the cathode to hold a high sulfur content of 70 wt% and a high sulfur loading of 8 mg cm^−2^, while attaining a high charge-storage capacity of 1087 mAh g^−1^ at a low electrolyte-to-sulfur ratio of 10 µL mg^−1^; these are the necessary parameters for developing a lithium-sulfur battery cathode with a high energy density. 

In support of this, the high-loading sulfur cathode demonstrated a superior areal capacity of 8.7 mAh cm^−2^ and an excellent energy density of 18.7 mWh cm^−2^, which are comparable to currently available lithium-ion cells (2–4 mAh cm^−2^ and 11 mWh cm^−2^). Moreover, our comparison analysis of the modification of the cell configuration and the material properties demonstrates that the modified carbon-nanofoam interlayers also improved the overall electrochemical characteristics. The major contribution resulted from the configuration design, namely, the adoption of a carbon nanofoam as the interlayer in the cell.

## Figures and Tables

**Table 1:** Chemical composition of carbon nanofoams.

Element (%)	Carbon Nanofoam	Mos_2_-Coated Carbon Nanofoam	Graphene-Coated Carbon Nanofoam
carbon	95.7	72.2	97.5
sulfur	0.0	6.5	0.0
molybdenum	0.0	19.6	0.0
oxygen	4.2	1.6	2.5
fluorine	0.1	0.1	0.0

**Table 2:** Performance comparison of the cells with each type of interlayer at the C/20 and C/10 rates.

	Carbon-Nanofoam Interlayer	MoS_2_-Coated Carbon-Nanofoam Interlayer	Graphene-Coated Carbon-Nanofoam Interlayer	Reference
C/20 analysis				
peak capacity [mAh g^−1^]	1381	1462	1484	558
reversible capacity [mAh g^−1^]	680	552	572	357
cycle life	200	100	100	100
retention rate	49%	38%	39%	64%
C/10 analysis				
peak capacity [mAh g^−1^]	1125	1109	1115	432
reversible capacity [mAh g^−1^]	500	413	428	222
cycle life	200	200	200	100
retention rate	44%	37%	38%	51%

**Table 3:** Comparative analysis of the battery performances and electrochemical characteristics of the high-loading sulfur cathode systems.

a	b	c	d	e	f	g	h
8	10	1057	8.4	42	200	C/10	This work
3	20	1085	3.3	95	50	C/5	[35]
3	20	867	2.6	90	200	1C	[35]
8.5	30	1150	9.8	79	100	C/2	[36]
8.5	30	952	8.1	75	200	C/5	[36]
12	20	1126	13.5	71	50	C/5	[37]
4	31	800	3.2	85	100	1C	[38]
6	31	600	3.6	75	100	1C	[38]
6	31	1059	6.3	78	100	1C	[38]
8	6.25	663	5.3	85	100	C/5	[39]
6.8	12	1000	6.8	88	10	C/20	[40]
6.3	13	1100	6.9	87	10	C/20	[40]
5	20	1104	5.5	72	80	C/10	[41]
6.8	20	1387	9.4	69	30	C/10	[41]
4	20	1000	4	70	70	C/5	[42]
4	10	1084	4.3	75	100	C/10	[43]
4	20	981	3.9	60	50	C/10	[44]
6	20	637	3.8	69	50	C/10	[44]
3.2	8	1150	3.7	45	120	C/10	[45]

a. sulfur loading [mg cm^−2^]; b. electrolyte-to-sulfur ratio [μL mg^−1^]; c. peak capacity [mAh g^−1^]; d. areal capacity [mAh cm^−2^]; e. capacity retention [%]; f. cycle life; g. cycling rate; and h. reference.

## Data Availability

Not applicable.
